# R-on-T–triggered ventricular fibrillation during acute anterior ST-elevation myocardial infarction

**DOI:** 10.1093/ehjcr/ytag342

**Published:** 2026-05-11

**Authors:** Göktuğ Savaş, Ali Nural, Selçuk Yazıcı, Sait Terzi

**Affiliations:** Department of Cardiology, Siyami Ersek Thoracic and Cardiovascular Surgery Training and Research Hospital, University of Health Sciences, No. 13 Tibbiye Street, 34668 Istanbul, Türkiye; Department of Cardiology, Siyami Ersek Thoracic and Cardiovascular Surgery Training and Research Hospital, University of Health Sciences, No. 13 Tibbiye Street, 34668 Istanbul, Türkiye; Department of Cardiology, Siyami Ersek Thoracic and Cardiovascular Surgery Training and Research Hospital, University of Health Sciences, No. 13 Tibbiye Street, 34668 Istanbul, Türkiye; Department of Cardiology, Siyami Ersek Thoracic and Cardiovascular Surgery Training and Research Hospital, University of Health Sciences, No. 13 Tibbiye Street, 34668 Istanbul, Türkiye

## Case description

A 63-year-old man presented with acute chest pain and diaphoresis. Blood pressure was 125/72 mmHg and heart rate 120 b.p.m. A 12-lead electrocardiogram (ECG) demonstrated sinus rhythm with marked ST-segment elevation in leads V1–V4 and reciprocal ST depression in the inferior leads, consistent with acute anterior ST-segment elevation myocardial infarction (*Figure 1*).

During ECG acquisition, a premature ventricular complex occurred on the descending limb of the T wave (the arrow in Figure 1), immediately triggering ventricular fibrillation, consistent with a classic R-on-T phenomenon. The abrupt transition from acute myocardial ischaemia to malignant ventricular arrhythmia was captured in real time on a standard 12-lead recording.

Immediate unsynchronized defibrillation restored sinus rhythm. Emergent coronary angiography revealed proximal left anterior descending artery occlusion, and successful percutaneous coronary intervention with drug-eluting stent implantation was performed. The patient recovered uneventfully and remained asymptomatic at 2-month follow-up.

The R-on-T phenomenon represents a premature ventricular complex occurring during the vulnerable phase of ventricular repolarization and is a well-recognized trigger of malignant ventricular arrhythmias. This image highlights the arrhythmogenic vulnerability of ischaemic myocardium and underscores the life-saving importance of rapid recognition and prompt defibrillation.^1^

**Figure 1:**
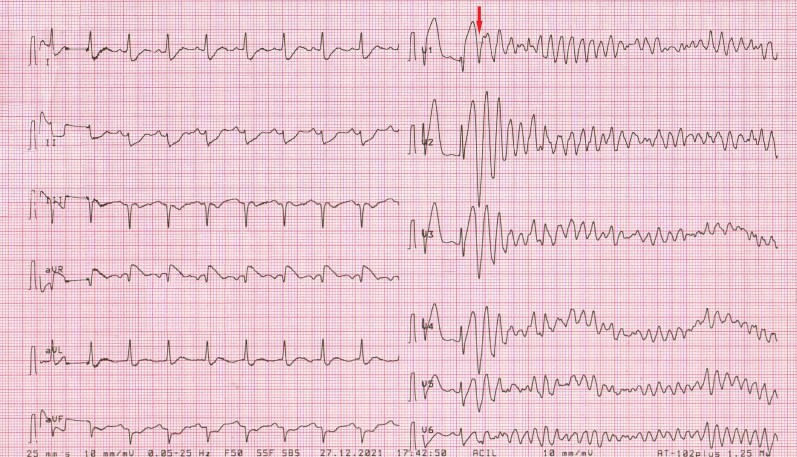
Twelve-lead electrocardiogram demonstrating anterior ST-segment elevation myocardial infarction with a premature ventricular complex falling on the descending limb of the T wave (arrow), immediately triggering ventricular fibrillation (R-on-T phenomenon).

## Data Availability

The data underlying this article are available within the article. No additional data are available.
